# Supplementary material for: A New Functional Magnetic Resonance Imaging Localizer for Preoperative Language Mapping Using a Sentence Completion Task: Validity, Choice of Baseline Condition, and Test–Retest Reliability
Source: Front Hum Neurosci. 2022 Mar 30;16:791577. doi: 10.3389/fnhum.2022.791577 (PMC9006995; doi:10.3389/fnhum.2022.791577)
Supplement: Supplementary file 1 [file Data_Sheet_1.pdf]

Supplementary Material

Table S1. Areas from the Brainnetome atlas (Fan et al., 2016) used to define language-related regions of interest

Language-related ROI	Areas from the Brainnetome atlas (Fan et al., 2016)
Pars triangularis of the IFG	IFG part 4 (rostral area 45), IFG part 3 (caudal area 45), IFG part 2 (inferior frontal sulcus)
Pars opercularis of the IFG	IFG part 5 (opercular area 44), IFG part1 (dorsal area 44)
pMFG	MFG part 2 (inferior frontal junction)
SMA	SFG part 4 (dorsolateral area 6), SFG part 5 (medial area 6)
pMTG	MTG part 1 (caudal area 21), MTG part 3 (dorsolateral area 37)
pSTG	STG part 4 (caudal area 22), STG part 2 (area 41/42)
Angular gyrus	IPL part 2 (rostrodorsal area 39), IPL part 5 (rostroventral area 39), IPL part 1 (rostrodorsal area 39)
Supramarginal gyrus	IPL part 4 (caudal area 40)

Note. IFG: inferior frontal gyrus, IPL: inferior parietal lobule, MFG: middle frontal gyrus, MTG: middle temporal gyrus, pMFG: posterior middle frontal gyrus, pMTG: posterior middle temporal gyrus, pSTG: posterior superior temporal gyrus, SFG: superior frontal gyrus, STG: superior temporal gyrus, SMA: supplementary motor area.

Table S2. Group-level activation clusters in the SYLL paradigm (paradigm with the syllable baseline), significant at three statistical thresholds (FWE: FWE correction for multiple comparisons at *a* = .05; AT: adaptive thresholding as implemented in Gorgolewski et al. (2012) at *a* = .05; Cluster: cluster correction at voxel-wise *a* = .001 and cluster-wise *a* = .05 FWE-corrected). Cluster localization was determined in the ICN_atlas toolbox (Kozák et al., 2017) based on the Brainnetome parcellation (Fan et al., 2016). Only clusters of ≥ 100 voxels are presented.

X	Y	Z	Peak *t*-value	Number of voxels	L/R	Cluster extent
					SYLL FWE	
-28	28	-2	10.47	442	L	Inferior frontal gyrus, orbital gyrus, insula
-6	14	60	10.7	139	L	Superior frontal gyrus
					SYLL AT	
-28	28	-2	10.47	893	L	Middle frontal gyrus, inferior frontal gyrus, orbital gyrus, insula, superior frontal gyrus
-58	-24	-2	8.70	228	L	Superior temporal gyrus, middle temporal gyrus, posterior superior temporal sulcus
-6	14	60	10.7	220	L	Superior frontal gyrus
					SYLL Cluster	
-28	28	-2	10.47	3037	L	Middle frontal gyrus, inferior frontal gyrus, orbital gyrus, precentral gyrus, superior temporal gyrus, middle temporal gyrus, insula, basal ganglia
12	-76	12	7.63	2532	L	Precuneus, medioventral occipital cortex, lateral occipital cortex, hippocampus;
					R	Precuneus, cingulate gyrus, medioventral occipital cortex, lateral occipital cortex
-6	14	60	10.70	1886	L	Superior frontal gyrus, middle frontal gyrus, cingulate gyrus;
					R	Superior frontal gyrus
-58	-24	-2	8.70	1671	L	Superior temporal gyrus, middle temporal gyrus, inferior temporal gyrus, posterior superior temporal sulcus
8	-72	-8	9.35	884	R	Medioventral occipital cortex, cerebellum
-42	4	56	8.83	817	L	Middle frontal gyrus, precentral gyrus, postcentral gyrus
-24	-98	10	7.45	730	L	Medioventral occipital cortex, lateral occipital cortex
40	28	-6	6.88	358	R	Inferior frontal gyrus, orbital gyrus, insula
6	10	20	8.05	303	R	Cingulate gyrus, basal ganglia
-48	-62	22	6.27	225	L	Posterior superior temporal sulcus, inferior parietal lobule
50	-8	34	5.59	186	R	Postcentral gyrus, precentral gyrus
-8	-28	-10	6.49	173	L	Thalamus

Note. L: left hemisphere, R: right hemisphere.

Table S3. Group-level activation clusters for the PW paradigm (paradigm with the syllable baseline), significant at three statistical thresholds (FWE: FWE correction for multiple comparisons at *a* = .05; AT: adaptive thresholding as implemented in Gorgolewski et al. (2012) at *a* = .05; Cluster: cluster correction at voxel-wise *a* = .001 and cluster-wise *a* = .05 FWE-corrected). Cluster localization was determined in the ICN_atlas toolbox (Kozák et al., 2017) based on the Brainnetome parcellation (Fan et al., 2016). Only clusters of ≥ 100 voxels are presented.

X	Y	Z	Peak *t*-value	Number of voxels	L/R	Cluster extent
					PW FWE	
-8	50	30	14.22	686	L	Superior frontal gyrus;
					R	Superior frontal gyrus
-52	22	12	13.98	673	L	Inferior frontal gyrus, orbital gyrus, insula
12	-70	-2	11.71	192	R	Medioventral occipital cortex
24	-80	-22	11.67	177	R	Cerebellum
-14	-46	0	9.70	123	L	Parahippocampal gyrus, cingulate gyrus, medioventral occipital cortex
-54	-10	-12	10.22	114	L	Superior temporal gyrus, middle temporal gyrus
					PW AT	
-8	50	30	14.22	861	L	Superior frontal gyrus;
					R	Superior frontal gyrus
-52	22	12	13.98	802	L	Inferior frontal gyrus, orbital gyrus, insula
12	-70	-2	11.71	253	R	Medioventral occipital cortex, cerebellum
-32	16	56	9.68	182	L	Superior frontal gyrus; middle frontal gyrus
-14	-46	0	9.70	167	L	Parahippocampal gyrus, cingulate gyrus, medioventral occipital cortex, hippocampus, cerebellum
-54	-10	-12	10.22	137	L	Superior temporal gyrus, middle temporal gyrus
					PW Cluster	
-8	50	30	14.22	22287	L	Superior frontal gyrus, middle frontal gyrus, inferior frontal gyrus, orbital gyrus, precentral gyrus, superior temporal gyrus, middle temporal gyrus, inferior temporal gyrus, fusiform gyrus, parahippocampal gyrus, posterior superior temporal sulcus, inferior parietal lobule, precuneus, insula, cingulate gyrus, medioventral occipital cortex, lateral occipital cortex, amygdala, hippocampus, basal ganglia, cerebellum
					R	Parahippocampal gyrus, cingulate gyrus, lateral occipital cortex, medioventral occipital cortex, fusiform gyrus, precuneus, hippocampus, cerebellum
52	28	4	7.91	1212	R	Inferior frontal gyrus, orbital gyrus, insula
-4	32	-14	6.71	672	L	Orbital gyrus, cingulate gyrus;
					R	Orbital gyrus
8	-8	52	8.42	664	R	Precentral gyrus, postcentral gyrus
38	-18	18	7.06	631	R	Inferior parietal lobule, postcentral gyrus, insula
-8	50	30	14.22	376	R	Superior frontal gyrus, fusiform gyrus, parahippocampal gyrus, precuneus, cingulate gyrus, medioventral occipital cortex, lateral occipital cortex, hippocampus

54	-2	-22	7.17	352	R	Superior temporal gyrus, middle temporal gyrus
-34	-22	20	6.47	331	L	Inferior parietal lobule, postcentral gyrus, insula
14	10	8	10.97	213	L	Basal ganglia, thalamus;
					R	Basal ganglia, thalamus

Note. L: left hemisphere, R: right hemisphere.

Table S4. Individual extent of activation in language-related regions of interest across participants, as a percentage out of the total number of voxels in the region based on the Brainnetome atlas parcellation (Fan et al., 2016). Percentages are presented for each paradigm (SYLL: syllable baseline vs. PW: pseudoword baseline) and statistical thresholding (FWE: FWE correction for multiple comparisons at *a* = .05; AT: adaptive thresholding as implemented in Gorgolewski et al. (2012) at *a* = .05; Cluster: cluster correction at voxel-wise *a* = .001 and cluster-wise *a* = .05 FWE-corrected).

	ROI	Min	25th percentile	Median	75th percentile	Max	IQR
PW FWE	IFG-tri	14.31	40.09	48.43	60.69	77.19	20.60
	IFG-oper	0.00	18.05	22.00	33.39	55.14	15.35
	pMFG	5.77	11.93	27.18	36.10	69.57	24.17
	SMA	0.00	0.00	0.00	0.00	2.85	0.00
	pMTG	0.00	6.24	10.79	19.42	39.43	13.18
	pSTG	0.00	0.66	2.62	5.42	15.32	4.75
	Angular	0.00	5.86	10.14	15.48	29.97	9.62
	Supramarginal	0.00	0.00	0.05	2.76	15.41	2.76
PW AT	IFG-tri	15.33	59.33	65.98	74.79	86.98	15.47
	IFG-oper	0.00	28.76	36.78	50.28	65.79	21.52
	pMFG	6.63	22.95	38.16	58.41	79.39	35.46
	SMA	0.00	0.00	0.86	2.10	4.49	2.10
	pMTG	0.00	12.03	22.70	28.45	48.61	16.42
	pSTG	0.09	2.36	8.07	13.17	21.19	10.81
	Angular	0.00	12.66	17.23	24.76	37.59	12.11
	Supramarginal	0.00	0.00	2.05	5.67	21.66	5.67
PW Cluster	IFG-tri	38.13	70.50	74.01	86.20	94.00	15.70
	IFG-oper	1.25	37.00	46.05	55.61	82.33	18.61
	pMFG	15.46	34.14	47.00	67.64	83.93	33.50
	SMA	0.00	0.05	2.50	5.32	12.91	5.26
	pMTG	0.00	16.18	24.69	40.21	52.15	24.03
	pSTG	1.01	6.38	13.26	16.65	25.50	10.28
	Angular	0.00	17.56	25.48	30.63	43.39	13.07
	Supramarginal	0.00	0.61	2.99	8.87	23.25	8.26
SYLL FWE	IFG-tri	6.65	22.16	30.52	41.97	69.25	19.81
	IFG-oper	0.00	15.95	26.82	44.30	57.39	28.35
	pMFG	0.00	6.87	23.44	40.49	70.55	33.62
	SMA	0.00	0.00	0.00	0.07	5.06	0.07
	pMTG	0.00	0.27	2.23	9.33	27.89	9.06
	pSTG	0.00	0.94	3.67	12.64	21.74	11.70
	Angular	0.00	1.27	2.30	4.10	26.46	2.83
	Supramarginal	0.00	0.00	0.00	0.33	12.61	0.33

SYLL AT	IFG-tri	0.00	17.38	37.21	53.28	71.19	35.89
	IFG-oper	0.00	16.07	28.20	46.77	60.15	30.70
	pMFG	0.37	6.44	15.40	42.52	75.71	36.08
	SMA	0.00	0.00	0.00	0.19	4.35	0.19
	pMTG	0.00	0.08	2.24	8.76	29.40	8.68
	pSTG	0.00	1.47	4.68	12.02	23.76	10.55
	Angular	0.00	1.31	3.49	10.32	27.48	9.02
	Supramarginal	0.00	0.00	0.00	0.23	21.10	0.23
SYLL Cluster	IFG-tri	15.05	51.92	58.96	64.61	86.15	12.69
	IFG-oper	0.00	44.61	56.52	66.29	85.46	21.68
	pMFG	4.42	13.90	43.13	64.42	90.92	50.53
	SMA	0.00	0.11	1.03	2.95	11.41	2.84
	pMTG	0.00	1.75	12.51	20.66	46.84	18.91
	pSTG	1.38	7.55	11.75	22.73	41.74	15.19
	Angular	0.00	6.54	10.24	18.65	37.74	12.11
	Supramarginal	0.00	0.00	0.52	3.15	21.57	3.15

Note. IQR: interquartile range, IFG-tri: pars triangularis of the inferior frontal gyrus, IFG-oper: pars opercularis of the inferior frontal gyrus, pMFG: posterior middle frontal gyrus, SMA: supplementary motor area, pMTG: posterior middle temporal gyrus, pSTG: posterior superior temporal gyrus, Angular: angular gyrus, Supramarginal: supramarginal gyrus.

Figure S1. Individual-level example: Significant language-related activation in the SYLL (panel A) and PW (panel B) paradigm in participant 03BT.

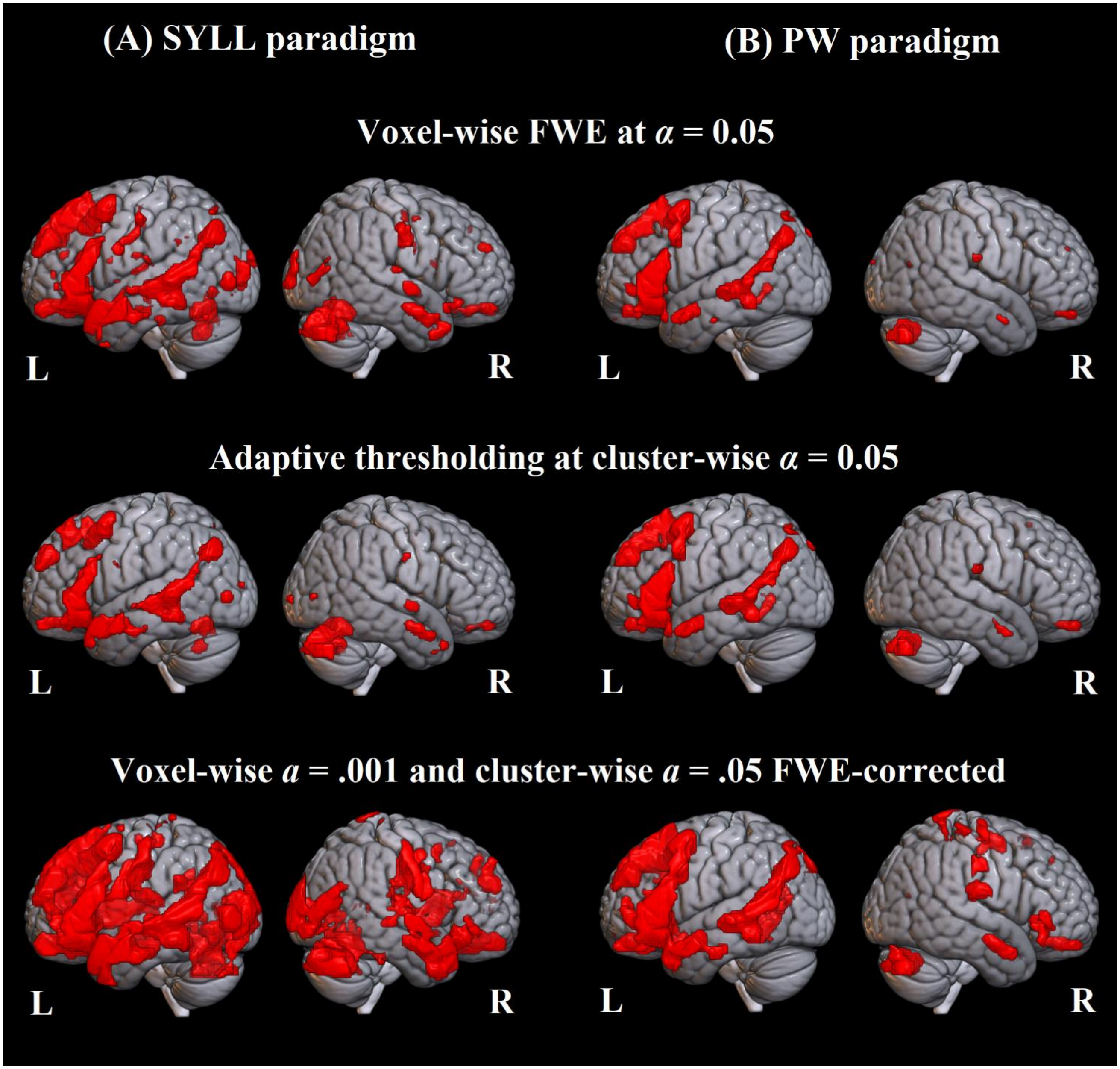

Note. Top: FWE correction for multiple comparisons at *a* = .05, middle: adaptive thresholding (AT) as implemented in Gorgolewski et al. (2012) at *a* = .05, bottom: cluster correction at voxel-wise *a* = .001 and cluster-wise *a* = .05 FWE-corrected.

Figure S2. Individual-level example: Significant language-related activation in the SYLL (panel A) and PW (panel B) paradigm in participant 12UV.

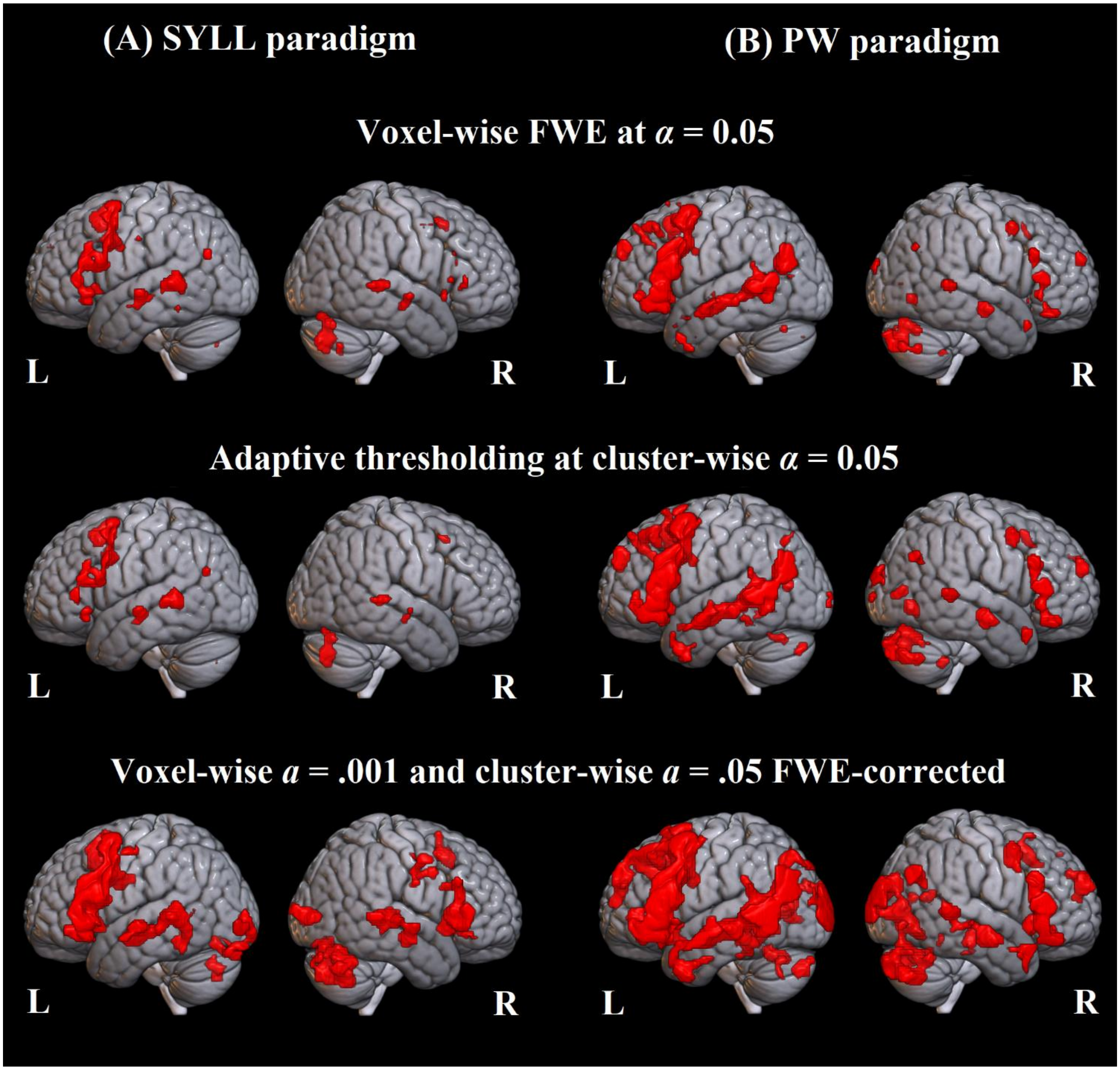

Note. Top: FWE correction for multiple comparisons at *a* = .05, middle: adaptive thresholding (AT) as implemented in Gorgolewski et al. (2012) at *a* = .05, bottom: cluster correction at voxel-wise *a* = .001 and cluster-wise *a* = .05 FWE-corrected.

Figure S3. Individual-level example: Significant language-related activation in the SYLL (panel A) and PW (panel B) paradigm in participant 21BA.

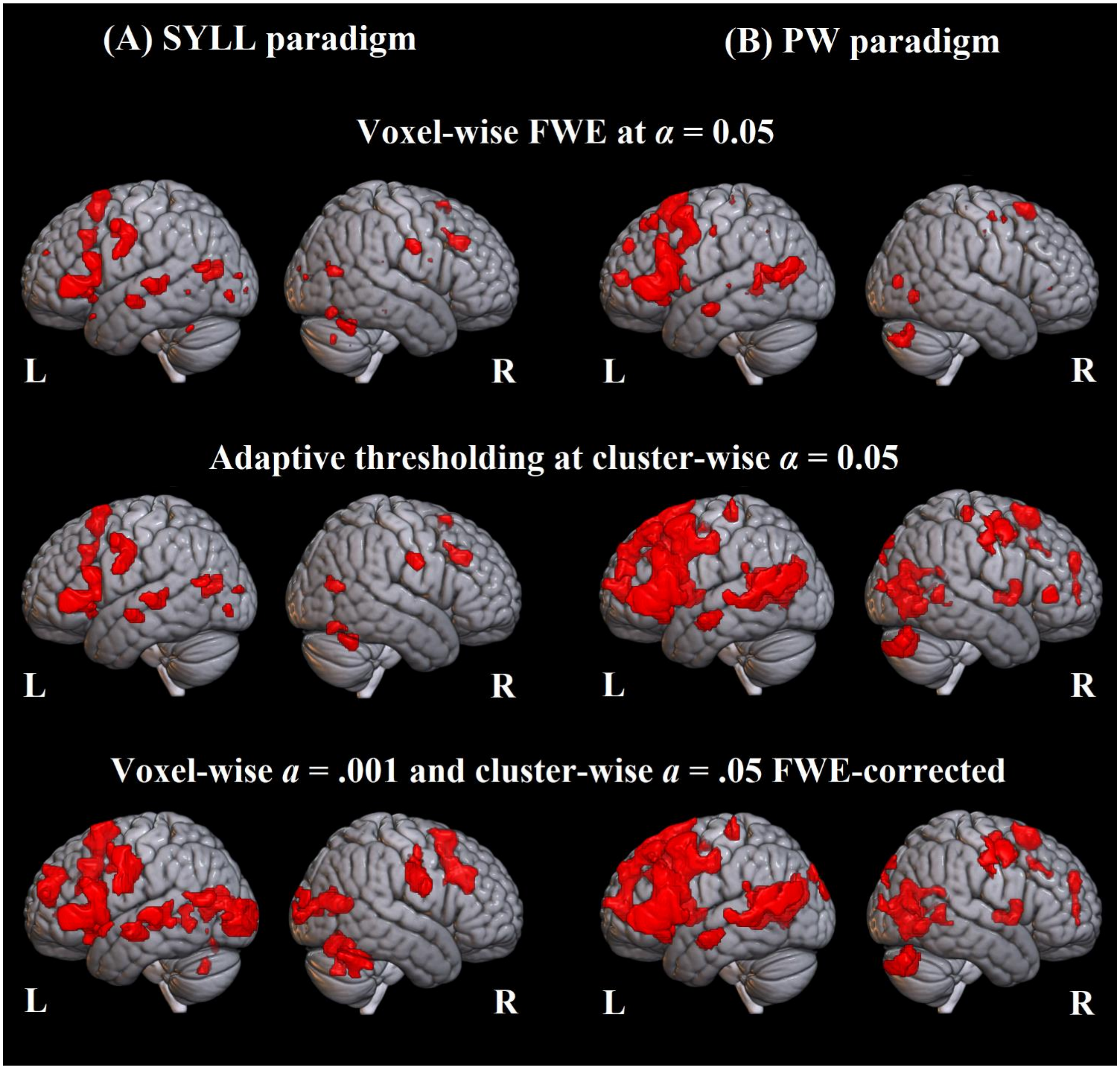

Note. Top: FWE correction for multiple comparisons at *a* = .05, middle: adaptive thresholding (AT) as implemented in Gorgolewski et al. (2012) at *a* = .05, bottom: cluster correction at voxel-wise *a* = .001 and cluster-wise *a* = .05 FWE-corrected.
